# Fault Detection in Axial Deformation Sensors for Hydraulic Turbine Head-Cover Fastening Bolts Using Analytical Redundancy

**DOI:** 10.3390/s26030801

**Published:** 2026-01-25

**Authors:** Eddy Yujra Rivas, Alexander Vyacheslavov, Kirill Gogolinskiy, Kseniia Sapozhnikova, Roald Taymanov

**Affiliations:** 1 Department of Metrology, Instrumentation and Quality Management, Empress Catherine II Saint Petersburg Mining University, Saint Petersburg 199106, Russia; 2 Department of Nuclear-Physics Research Methods, Saint Petersburg State University, Saint Petersburg 199034, Russia; 3 Laboratory for Metrological Maintenance of Computerized Sensors and Measuring Systems, D.I. Mendeleyev Institute for Metrology, Saint Petersburg 199005, Russia; k.v.s@vniim.ru (K.S.); r.e.taimanov@vniim.ru (R.T.)

**Keywords:** fault detection, analytical redundancy, Kalman filter, monitoring system, Francis turbine, bolts deformation

## Abstract

This study proposes an analytical redundancy method that combines empirical models with a Kalman filter to ensure the reliability of measurements from axial deformation sensors in a turbine head-cover bolt-monitoring system. This integration enables the development of predictive models that optimally estimate the dynamic deformation of each bolt during turbine operation at full and partial load. The test results of the models under conditions of outliers, measurement noise, and changes in turbine operating mode, evaluated using accuracy and sensitivity metrics, confirmed their high accuracy (*Acc* ≈ 0.146 µm) and robustness (*S_A_* < 0.001). The evaluation of the models’ responses to simulated sensor faults (offset, drift, precision degradation, stuck-at) revealed characteristic residual patterns for faults with magnitudes > 5 µm. These findings establish the foundation for developing a fault detection and isolation algorithm for continuous monitoring of these sensors’ operational health. For practical implementation, the models require validation across all operational modes, and maximum admissible deformation thresholds must be defined.

## 1. Introduction

High-hydroelectric-power plants comprise several hydroelectric units. Each hydroelectric unit primarily consists of a hydro generator and a hydraulic turbine (hereafter referred to as simply the turbine). During operation, the turbine head-cover experiences significant static and dynamic loads. According to the study presented in [1], the hydraulic flow can generate axial forces acting on the head-cover during the startup phase, reaching values up to 33 times its weight. During load rejection tests, these forces exceed 10 times the total weight of the head-cover and rotating shaft, as reported in [2].

These forces induce significant mechanical stresses and deformations both in the head-cover [3,4], and in the bolts that fasten the cover to the turbine stator (the casing component) [5,6]. Furthermore, phenomena such as cavitation [7], vortex formation [8], and water hammer [9], which occur during turbine operation, generate vibrations that not only accelerate the wear of mechanical components but also impose additional mechanical loads on the fastening bolts [10,11,12,13]. Due to these substantial loads, the fastening bolts are among the most critical components of a hydroelectric unit.

Numerical studies on the deformation of fastening bolts have focused on analyzing both static (preload) and dynamic (preload and hydraulic force) loads that cause bolt deformation. Research [4,14,15] indicates that the greatest deformation in a turbine occurs in the inner central part of the head-cover, while the deformation of the bolts around its perimeter is lower. As noted in [5,16,17], the bolts are subjected not only to tensile but also to bending stresses, leading to stress concentration at the transition area between the threaded surface and the smooth surface of the bolt. It has also been demonstrated that bolt deformation varies depending on the head-cover structure along its perimeter, being greater in covers with a double-flange design [18].

There are relatively few methods reported in the literature for measuring mechanical stress or deformation in fastening bolts. A review of systems for measuring these quantities [19] notes that, although they enable quantitative evaluation of their behavior, only the PTK KM-Delta system ensures measurement reliability through its sensor fault detection function. However, this function is limited and cannot detect faults related to changes in precision, offset, or measurement drift.

Fault detection and diagnosis in measurement systems have been the subject of extensive research employing diverse methods and technologies coexist. Digital twins create an advanced virtual model of the physical system, enabling the continuous detection of anomalies by comparing expected (simulated) behavior with actual measured performance [20,21]. Graph Neural Networks, based on deep learning, model relationships and dependencies between sensors and components to infer the system’s state and thus diagnose faults from its interconnection structure [22,23,24,25]. For their part, systems based on fuzzy logic effectively handle imprecision and expert knowledge through linguistic rules, being especially useful in contexts where the system is not known with mathematical exactitude or where variables lack clearly defined limits [26,27,28]. An emerging approach uses trained large language models to acquire industrial reasoning capabilities for complex tasks like fault detection [29,30,31]; however, it requires extensive industrial datasets.

On the other hand, the reliability of measurement results is inextricably linked to the outcomes of metrological conformity assessment, which, according to ISO 10012 [32,33], encompasses calibration and verification, any necessary adjustment and repair followed by recalibration, comparison with the metrological requirements for the intended use of the equipment, as well as sealing and labeling. However, traditional calibration and verification methods face significant challenges in critical sectors—such as oil and gas, aerospace, nuclear, and power generation [34,35,36,37,38,39]. These limitations are primarily due to the complexity of disassembling the measurement systems and the impact of harsh operating conditions, which hinder the execution of traditional calibration and verification procedures. In view of these constraints, alternative approaches are being investigated, such as self-calibration (SC) [40,41,42], on-line calibration monitoring (OLM) [43,44,45,46,47], metrological self-check (MSC) in intelligent sensors/systems as per the Russian standard GOST R 8.734 [48,49,50], and self-validating sensor (SEVA) technology [51,52,53]. Each approach is based on utilizing additional information derived from structural, temporal, or functional (analytical) redundancy.

Given the critical importance of ensuring the structural integrity of the hydraulic turbine head-cover fastening bolts, this paper proposes a method based on analytical redundancy to ensure the reliability of measurement results provided by the sensors of a system that monitors the axial deformation of these bolts. The method is aimed at early fault detection in the sensors of the measurement system.

This study is organized as follows. Section 2 presents the measurement system used to obtain the measurement data analyzed in this work, as well as the proposed method. Section 3 is devoted to the procedure for constructing the predictive models required for implementation of the method. Section 4 describes the procedure used for their validation. Section 5 defines the metrics used to assess their performance. Section 6 presents and discusses the obtained results. Finally, Section 7 formulates the main conclusions of the study and outlines directions for future research.

## 2. Measurement System and Method

The data for this study were obtained from axial deformation measurements of a Francis turbine head-cover bolts. The measurements were performed using the system described in Section 2.1. All calculations, simulations, tests, and data visualization were carried out in the MATLAB R2024a software environment.

### 2.1. Axial Deformation Measurement System

**Figure 1:**
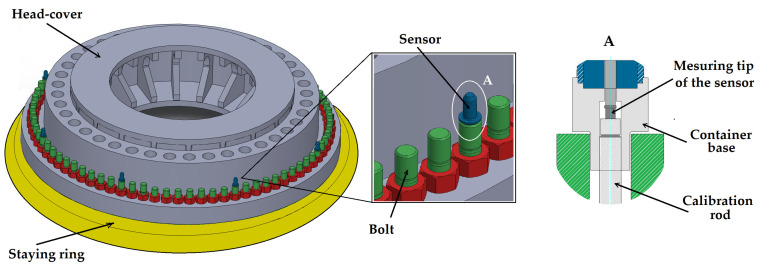
Scheme of the sensors mounting on the fastening bolts: A—shows the internal view of the sensor mounting on the bolt (compiled by the authors).

The flange of the Francis turbine head-cover is fastened to the turbine’s staying ring flange using 80 bolts, uniformly distributed along its perimeter. The measurement system is designed to measure the axial deformation of the bolts by measuring the displacement of the bolt ends relative to calibration rods installed within eight of them (one every tenth bolt), as shown in Figure 1.

As illustrated in Figure 1, contact linear displacement transducers (hereinafter referred to as sensors) convert the bolt displacement relative to the calibration rods—fixed to the turbine’s staying ring—into an electrical signal via an intermediate optical conversion stage. This signal is digitized and transmitted to a processing unit, which provides further signal transmission and displays the measurement results. Table 1 briefly summarizes the main characteristics of the measurement system.

Using the described system, axial deformation measurements of eight bolts (in μm) were recorded over four days distributed throughout a month. Head water pressure (in m) and generated power (in MW) were simultaneously recorded. These data correspond to the hydroelectric unit’s power grid connection stage [19]. Although the sampling time was 1 s, a representative subsample of 14,000 data points per day was selected for analysis, clearly capturing bolt deformation variations during grid connection events.

**Figure 2:**
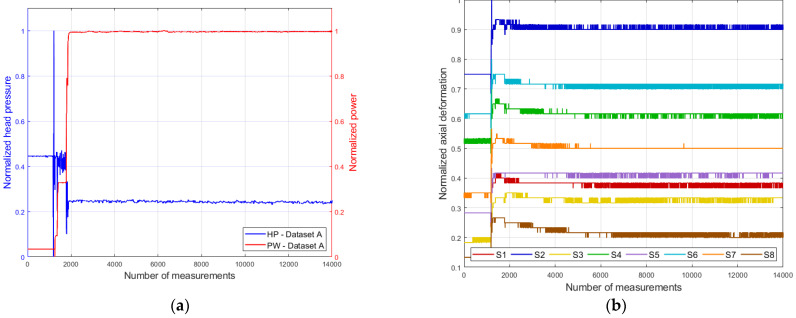

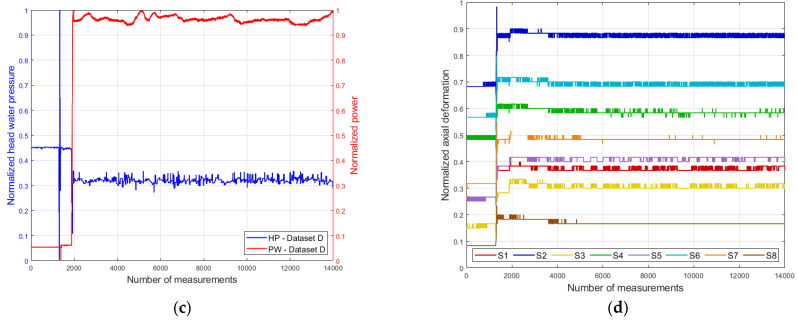
Data of generated power (PW), head pressure (HP), and axial deformation of the fastening bolts (S1–S8): (**a**,**b**)—dataset D1; (**c**,**d**)—dataset D4 (compiled by the authors).

The data were grouped into four datasets (D1, D2, D3, and D4), each corresponding to a measurement day. The first three datasets correspond to the hydroelectric unit operating at full load, while dataset D4 corresponds to operation at 70% load. Figure 2 presents the variations in generated power, head water pressure (head pressure), and axial deformation of the bolts corresponding to datasets D1 and D4, depending on the hydroelectric unit’s operating mode (full or partial load). Datasets D2 and D3 are not included in the figure, but they exhibit trends similar to those of dataset D1. The recorded values of the measured quantities were normalized according to Formula (1).

(1) $$x_{normj}=\frac{x_{measj}-x_{min}}{x_{max}-x_{min}}$$

where: $x_{normj}$—normalized value for the *j*-th measurement; $x_{measj}$—*j*-th measured value; $x_{max}$—maximum value; $x_{min}$—minimum value.

It should be noted that each measured quantity has its own individual maximum and minimum values. In the case of the bolts, the maximum and minimum values were selected from the data of the eight measurement channels.

### 2.2. Analytical Redundancy Method

**Figure 3:**
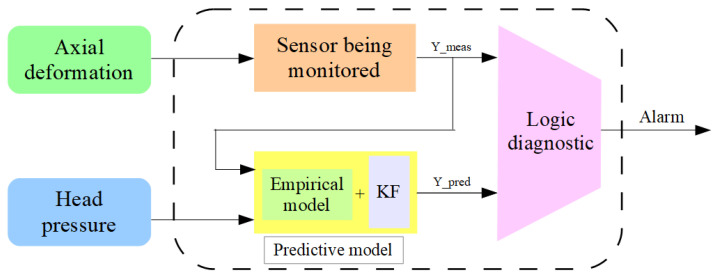
Schematic of the proposed analytical redundancy method to ensure reliability of measurement results in axial bolt deformation monitoring (compiled by the authors based on [52]).

The method for ensuring the reliability of measurement results from the sensors in the system described in Section 2.1 is based on the use of analytical redundancy. This method refers to redundancy achieved not by duplicating physical components or sensors (structural redundancy), but through a mathematical model and analysis techniques that enable the generation of additional information for detecting faults in sensors or measurement systems [55]. The model required for this is created based on physical principles or empirical data [52,56]. The reliability of measurement results is ensured by calculating and analyzing the residuals between the measured values (*Y_meas_*) and those predicted by the model (*Y_pred_*). These residuals are used as indicators for detecting potential deviations or faults in sensor operational health [53,57,58]. Figure 3 presents a schematic of the analytical redundancy implementation proposed in this study.

Since the axial deformation differs for each bolt as observed in Figure 2d, we propose developing an individual model (hereinafter referred to as the predictive model) for each sensor.

Our analytical redundancy approach integrates empirical models to which a Kalman filter (KF) is applied to design highly robust predictive models. Unlike standard approaches, this configuration produces models capable of estimating axial deformation during both full load and partial load operation of the hydraulic unit. This feature overcomes the limitations of established OML methods using purely empirical models, for example, based on auto-associative kernel regression [44], whose predictions are unreliable when the operating mode changes. Additionally, the generated residuals contain patterns about the type of fault that occurred, unlike methods based on non-redundant variables that do not allow its identification [45].

## 3. Methodology for Developing Predictive Models

**Figure 4:**
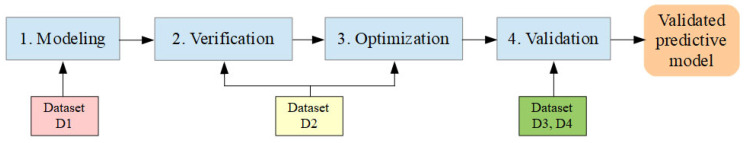
Schematic of analytical redundancy for ensuring the reliability of measurement results obtained from a sensor (compiled by the authors).

Figure 4 presents the methodology for developing predictive models, which includes four stages: modeling, verification, optimization, and validation. Specific datasets were used for each stage: dataset D1 was applied for estimating the parameters of empirical models; dataset D2 was used for empirical model verification and optimization of axial deformation estimation for the bolts; and dataset D3 for predicted models’ validation. Data from dataset D4 were used in one of the validation procedures for the predictive models.

### 3.1. Modeling

During the modeling stage, empirical models predicting the deformation dynamics of the fastening bolts were identified for each sensor (eight models in total) based on dataset D1, using the System Identification Toolbox in MATLAB. Additionally, the prediction uncertainty was quantitatively estimated for each obtained model.

#### 3.1.1. Model Structure and Parameters

The identification process involved determining a mathematical structure that models the effect of head pressure changes (input signal) on the axial deformation of a fastening bolt (output signal). The selected model structure is presented as a transfer function (2) of the complex variable ‘*s*’ (Laplace variable).

(2) $$G_{i\left(s\right)}=\frac{Kp_{i}\left(s+Tz_{i}\right)}{\left(Tp1_{i}+s\right)\times \left(Tp2_{i}+s\right)};\;\;i=1,\ldots \ldots ,8$$

where $G_{i\left(s\right)}$ is the transfer function describing the deformation dynamics of the *i*-th bolt; *Kp*, *Tz*, *Tp*1, and *Tp*2 are the parameters characterizing the dynamic properties of the corresponding bolt under head pressure influence; *i* denotes the specific model for each sensor.

The second-order structure was selected based on the bolt deformation behavior in response to head-pressure changes, as observed when plotting datasets D1–D3. This behavior exhibits the characteristic step response of a second-order system. The adaptive Gauss–Newton method was selected to estimate the *Gi*(*s*) parameters using MATLAB’s Toolbox, as it yields a lower mean squared error compared to other available methods, achieving superior fit to the validation data.

The transfer function *Gi*(*s*) constitutes an empirical model approximating the “head pressure–deformation” dynamics for the fastening bolt. This model serves, firstly, to verify the adequacy of the bolt deformation dynamics description and, secondly, as the basis for the subsequent stage focused on optimizing the bolt deformation estimation.

#### 3.1.2. Model Uncertainty Estimation

The parameters of the empirical models *Gi*(*s*) were estimated based on data obtained from sensors installed on eight bolts of the turbine head-cover and a head pressure sensor. These sensors possess their own inherent error. Furthermore, during measurement, factors such as measurement noise, external interference, and discretization could have affected the measurement accuracy [59,60,61]. The aforementioned factors directly impact the representativeness and quality of the datasets used for models’ estimation. Therefore, quantifying the uncertainty associated with each empirical model is necessary. Methods for uncertainty estimation—the analytical method and the Monte Carlo method—are detailed in the Guide to the Expression of Uncertainty in Measurement (GUM), according to the normative documents JCGM 100 [62] and JCGM 101 [63].

The models’ uncertainty using the analytical method was estimated based on the mathematical structure of *Gi*(*s*) (Formula (2)), initially assuming that the parameters *Kpi*, *Tzi*, *Tp1i*, and *Tp2i* are uncorrelated. Sensitivity coefficients were computed using the partial derivatives of *Gi*(*s*) with respect to each parameter. The variance in each parameter was determined from the diagonal of the covariance matrix obtained during the parameters’ estimation using the Adaptive Gauss–Newton method. The square root of these variances represents the individual uncertainty of each parameter. Subsequently, the combined standard uncertainty for each model was calculated. Finally, the expanded uncertainty *U_AM_* with a confidence level of 95% (k = 2) was determined.

Model uncertainty estimation using the Monte Carlo method was performed through *N* stochastic simulations of the parameters *Kpi*, *Tzi*, *Tp*1*i*, and *Tp*2*i*. For this purpose, the parameter values, initially estimated by the Adaptive Gauss–Newton method, were assigned normal probability distributions. This choice is justified by the fact that the estimation method used provides parameter covariance matrices, whose calculation assumes normality of the estimates. The means of each distribution corresponded to the nominal parameter values, and the standard deviation (σ) was calculated as the square root of the elements on the diagonal of the covariance matrix obtained during the parameter estimation. The variation limits for each parameter were set at ±3σ from the nominal value, encompassing approximately 99.7% of the distribution. Based on these distributions, *N* random parameters sets were generated, which allowed for the construction of *N* empirical models for each sensor. These models were applied to the input signal (head pressure), resulting in *N* model responses. The standard deviation of this response was taken as the models’ standard uncertainty. To determine the expanded uncertainty *U_MC_*, the standard deviation was multiplied by a coverage factor of k = 2, corresponding to a 95% confidence level.

### 3.2. Verification

During the verification stage, dataset D2 was used to assess the systematic error (bias) of the predictions performed by the empirical models. Bias for *N* measurements is calculated as the average of the differences between each measured value and the true (reference) value [64]. Since it is necessary to analyze the bias of each axial deformation prediction, measured values from dataset D2 were taken as reference. Thus, in this work, bias was calculated using Formula (3) to determine whether the predicted values from the empirical models overestimate or underestimate the measured values.

(3) $$Bias\approx \;\frac{1}{N}\overset{N}{\underset{j=1}{\sum }}\left({\hat{x}}_{j}-x_{j}\right)$$

where *N* is the number of measurements, $x_{j}$ is the measured value for the j-th measurement, and ${\hat{x}}_{j}$ is the corresponding predicted value.

Additionally, it is analyzed whether the empirical models *Gi*(*s*) reproduce the axial deformation dynamics, specifically its transient and steady-state responses. The transient state is the time interval during which the system responds to head pressure changes before reaching equilibrium. When the head pressure no longer changes over time, the system reaches equilibrium or steady-state condition.

### 3.3. Optimization

During the optimization stage, the accuracy of estimating the axial deformation of the fastening bolts was improved using previously verified empirical models. For this purpose, a recursive Kalman filter algorithm was applied to the representation of the empirical model *Gi*(*s*) in state space. According to theory [65,66] the Kalman filter enables the estimation of the state of a dynamic system under conditions where measurements contain noise and errors, optimizing estimation accuracy through a recursive process of prediction and correction. The application of the Kalman filter is widespread, covering various fields from engineering [67,68] to economics [69,70,71,72].

The representation of *Gi*(*s*) in state-space form was obtained by transforming it into the controllable canonical form of second order, as described in [73]. In continuous time, this representation takes the form of Equation (4):

(4) $${\dot{x}}_{i}=\;A_{i}x_{i}+B_{i}u;\;\;\;where\;\;\;\;A_{i}=\left[\begin{array}{cc} -\frac{Tp1_{i}+Tp2_{i}}{Tp1_{i}\times Tp2_{i}} & 1\\ -\frac{1}{Tp1_{i}\times Tp2_{i}} & 0 \end{array}\right],\;\;\;B_{i}=\left[\begin{array}{c} \frac{Kp_{i}\times Tz_{i}}{Tp1_{i}\times Tp2_{i}}\\ \frac{Kp_{i}}{Tp1_{i}\times Tp2_{i}} \end{array}\right]\;y_{i}=C_{i}x_{i}+D_{i}u;\;\;where\;\;\;\;C_{i}=\left[\begin{array}{c} 1\\ 0 \end{array}\right],\;\;\;D_{i}=0$$

where $x_{i}$ is the state vector of the system, ${\dot{x}}_{i}$ is the state vector derivative, u is the input vector, y is the output vector, $A_{i}$ is the system matrix, $B_{i}$ is the input matrix, $C_{i}$ is the output matrix, $D_{i}$ is the feedthrough matrix and *i* denotes the correspondence to each estimated model.

Subsequently, the discretization of Equation (4) was performed using MATLAB’s *c*2*d* function and a sampling rate of 1 s, which converts the continuous-time model to discrete-time. Thus, the state-space representation of *Gi*(*s*) is given by (5), enabled the development of predictive models S1–S8.

(5) $$x_{k+1}=Fx_{k}+Gu_{k}+w_{k}\;z_{k}=Hx_{k}+v_{k}$$

where $x_{k+1}$ is the state vector of the system at time k + 1, $x_{k}$ is the state vector at the previous time *k*, $u_{k}$ is the control input vector, and $z_{k}$ is the measurement vector of the quantity that the model attempts to estimate indirectly through the system state. *F*—is the state transition matrix, *G*—is the input matrix, and *H*—is the observation matrix. Matrices *F*, *G*, and *H* describe the system dynamics and the relationship between the state and the measurement. The terms $w_{k}$ and $v_{k}$ correspond to process noise and measurement noise, respectively, both modeled as Gaussian random variables with zero mean and covariances *Q* and *R*.

It should be noted that process noise $w_{k}$ models external and internal disturbances and errors in the system dynamics not accounted for in the idealized model. Meanwhile, measurement noise $v_{k}$ reflects random measurement errors and disturbances arising during data collection about the system state.

For optimal state estimation, the Kalman filter algorithm operates in two stages:

1. Prediction—the algorithm determines a preliminary (a priori) state estimate based on the system’s dynamic model and its uncertainty (covariance) before obtaining the current measurement.
  (6) $${\hat{x}}_{k,\;\;k-1}=F{\hat{x}}_{k,k}+Gu_{k-1}\;P_{k,k-1}=FP_{k,k}F^{T}+Q$$
  where ${\hat{x}}_{k,\;k-1}$ represent the a priori state estimate and $P_{k,k-1}$ is the uncertainty of the prediction (before measurement), ${\hat{x}}_{k,\;k}$ and $P_{k,k}$ correspond to the a posteriori estimate and covariance (after the measurement), and *Q* is the process noise covariance matrix.
2. Correction—after obtaining the current measurement, the previous prediction is corrected by combining it with the current measurement. For this correction, the Kalman gain is calculated, which determines how much the prediction should be adjusted based on the uncertainty of the measurement and the prediction. Subsequently, the state estimate ${\hat{x}}_{k,\;k}$ and the error covariance $P_{k,k}$ are updated.
  (7) $$K_{k}=P_{k,k-1}H_{k}^{T}{\left(H_{k}P_{k,k-1}H_{k}^{T}+R_{k}\right)}^{-1}\;{\hat{x}}_{k,k}={\hat{x}}_{k,k-1}+K_{k}\left(z_{k}+H_{k}{\hat{x}}_{k,k-1}\right)\;P_{k,k}=\left(I-K_{k}H_{k}\right)P_{k,k-1}{\left(I-K_{k}H_{k}\right)}^{T}+K_{k}R_{k}K_{k}^{T}$$
  where $K_{k}$ is the Kalman gain coefficient, *H*—is the observation matrix, *R*—is the measurement noise covariance matrix, and *I*—is the identity matrix.

Therefore, the stages complement each other: the prediction forms the basis, while the correction refines the estimate by incorporating new data. These two stages are repeated at each step of the filter’s operation, providing optimal smoothing and state estimation of the system.

Additionally, the diagonal elements of the covariance matrix *Q*, which quantify the prediction uncertainty of the empirical models *Gi*(*s*), were calculated from the maximum modeling uncertainty estimated via analytical (*U_AM_*) and Monte Carlo (*U_MC_*) methods. Since both uncertainties can be understood as the standard deviation multiplied by a coverage factor k = 2 (95% confidence level), the variance was calculated using σ^2^ = (*U*/k)^2^.

The variation in the axial deformation sensor measurements reflects the associated measurements uncertainty. Therefore, the element of the covariance matrix *R*, which quantifies sensor measurement error uncertainty, was adjusted to the maximum steady-state variance across the eight sensors, ensuring a conservative estimate of the actual hydraulic unit operation.

## 4. Validation of Predictive Models

The validation of predictive models is carried out by evaluating their performance under the influence of various factors, combined with an analysis of residual behavior during sensor faults.

### 4.1. Model Performance Under Different Operating Conditions

To analyze the performance of the models under different operating conditions, testing methods were developed that account for the following factors: the presence of outliers, measurement noise, and change in the operating mode of the hydroelectric unit (HU).

Outliers are defined as data points that significantly deviate from the system’s expected behavior. They may arise from sensor errors, data transmission issues, or rare physical phenomena [74]. For simulation purposes, ten outlier values were generated using Formula (8), based on the three-sigma rule.

(8) $$x_{outlier}>x_{meas}+3\sigma$$

where $x_{meas}$ is the measured value and *σ* is the maximum standard deviation calculated from the deformation measurements of the eight bolts.

The value of *σ* was determined based on measurements taken once the system reached equilibrium. These outliers were randomly added to the bolt axial deformation data in the D3 dataset.

On the other hand, measurement noise consists of random, unpredictable errors that distort the values of a system’s input and output signals. It is present in any real-world data acquisition process and is a continuous stochastic process. The simulation of measurement noise was performed by adding random perturbations, with an amplitude not exceeding the ±2σ interval, to the bolt axial deformation data in the D3 dataset.

Change in operating mode refers to the operation of the HU at various load levels, determined by the needs of the power system. The need for such evaluation arises because the parameters of the empirical models were estimated based on dataset D1, collected during full load operation of the HU. Evaluating the predictive capability of the models exclusively in this mode would lead to an overestimated, and therefore, incorrect assessment of their adequacy. This is because, in real-world conditions, the HU operates not only at full load but also, for example, at 70% of its nominal load. For this test, the dataset D4, which reflects HU operation at partial load (70%), was used.

The conducted tests allowed for the evaluation of prediction accuracy, model robustness, and their ability to forecast under changing HU operating modes. To quantitatively assess the predictive models’ performance, accuracy, sensitivity, and detectability metrics were used, which are described in detail in Section 5.

### 4.2. Model Response to Sensor Faults

As noted in Section 2.2, analytical redundancy enables the generation of additional information to detect sensor faults, which constitute a potential cause of their failure—terms clearly differentiated in the ISO 13372 [75] and ISO/IEC/IEEE 24765 [76] standards. This subsection analyzes the response of the predictive models during simulated faults.

Based on a literature analysis, Table 2 was developed, summarizing fault types, their definitions, and causes.

The analysis of model response to sensor faults is based on calculating the residual vector that arises from comparing measured and predicted values (Formula (9)).

(9) $$r_{j}=x_{j}-{\hat{x}}_{j}$$

where $r_{j}$ is the residual vector, $x_{j}$ is the measured value, ${\hat{x}}_{j}$ is the corresponding predicted value, and *j* is the measurement index.

The fault types described in Table 2 were simulated by injecting anomalous axial deformation values into dataset D3. Each fault was artificially simulated using a specific mathematical model described in [79].

The Bias Fault was simulated using the following expression, which applies a constant offset to the measured value:

(10) $$x_{bias}=\left(1+bias\%\right)\times x_{meas}$$

where $x_{bias}$ is the biased sensor reading, $x_{meas}$ is the measured value, and *bias*% is the percentage of added offset relative to the system’s absolute error.

To simulate drift, a linear model was used that gradually changes the measured value:

(11) $$x_{drift}=\left(1+\frac{z-z_{init}}{z_{end}-z_{init}}drift\%\right)\times x_{meas}$$

where $x_{drift}$ is the drifted sensor reading, *z* is the current measurement index, $z_{init}$ is the measurement index at the fault initiation moment, $z_{end}$ is the measurement index at the final simulation moment, and *drift*% is the percentage of added drift relative to the system’s absolute error.

Precision degradation was simulated by adding variance with increasing amplitude to the measurement results. To simulate progressive degradation, three levels were applied, corresponding to the sensor’s scale division: 0.5 µm, 1.0 µm, and 1.5 µm. The simulation was performed using the formula:

(12) $$x_{prec}=\;x_{meas}\pm var$$

where $x_{prec}$ is the sensor reading with degraded precision and *var* is Gaussian noise with zero mean and a variance equal to the specified degradation level.

The stuck-at fault was simulated by fixing the sensor readings to the value reached at the moment of fault initiation. This value, remained constant until the end of the measurements, regardless of changes in the actual axial deformation, and is expressed as:

(13) $$x_{stuck}=\;x_{meas}\left(z_{init}\right)$$

where $x_{stuck}$ is the sensor reading with a stuck value and $z_{init}$ is the measurement index at the moment of fault initiation.

The goal of the analysis is to identify patterns in residual behavior (such as minima, maxima) to determine the time of occurrence, type, and location of the fault. It should be noted that under ideal conditions (in the absence of sensor faults), the values of the residual vector $r_{j}$ tend toward zero, and their deviations form the basis for diagnostics.

## 5. Performance Metrics

The performance metrics applied in this study are based on scientific publications concerning the evaluation of empirical models used for predicting operational parameters of measurement systems and detecting sensor faults [85,86,87].

### 5.1. Accuracy

The Accuracy (*Acc*) metric assesses the quality of model predictions by comparing them with measured values [86]. In this study, the *Acc* metric was defined by calculating the statistical indicator—Mean Absolute Error (MAE) [87]. MAE indicates, on average, how much the predictions differ from the measured values in the same units. The formula for calculating the accuracy metric is presented below.

(14) $$Acc=\frac{1}{N}\overset{N}{\underset{j=1}{\sum }}\left|x_{j}-{\hat{x}}_{j}\right|$$

where *N* is the number of measurements, $x_{j}$ is the measured value for the j-th measurement, and ${\hat{x}}_{j}$ is the corresponding predicted value.

It should be noted that a lower *Acc* value indicates greater model accuracy, as the predicted values are closer to the measured values and, therefore, more reliable.

### 5.2. Sensitivity

In addition to accuracy, an assessment of model robustness is required. In accordance with ISO 15289 [88] and ISO 16336 [89] standards, system robustness refers to its ability to maintain correct and reliable operation in the presence of unacceptable input data, adverse environmental conditions, or external disturbances. In this context, sensitivity is a key metric that quantitatively characterizes one aspect of robustness—the degree to which changes in input data affect the model output.

In this work, to assess the sensitivity ($S_{A}$) of the models, the auto-sensitivity metric described in [86] is used. This metric evaluates the model’s ability to maintain prediction accuracy under deviations (*dev*) of input values caused by, for example, measurement noise or outliers. The $S_{A}$ value ranges from 0 to 1. A value close to zero indicates low model sensitivity to deviations, which is preferable. In this case, the residual between measured and predicted values primarily reflects the prediction error, facilitating its detection. The $S_{A}$ metric is defined by the following formula:

(15) $$S_{A}=\;\frac{1}{N}\overset{N}{\underset{j\;=1}{\sum }}\left|\frac{{\hat{x}}_{j}^{dev}-{\hat{x}}_{j}}{x_{j}^{dev}-x_{j}}\right|$$

where *N* is the number of measurements, $x_{j}$ is the j-th measured value without deviation, ${\hat{x}}_{j}$ is the corresponding model prediction for this value, $x_{j}^{dev}$ is the j-th measured value with deviation, and ${\hat{x}}_{j}^{dev}$ is the corresponding model prediction for this value with deviation.

### 5.3. Detectability

This metric quantifies the smallest anomaly or fault that can be detected by a model. In this study, the detectability of the eight models is evaluated using the Error Uncertainty Limit Monitoring ($D_{EULM}$) method [85]. This method detects faults by analyzing the uncertainty of prediction errors relative to a predefined tolerance. A sensor is considered faulty when the uncertainty interval of its prediction exceeds this tolerance. The EULM method is particularly useful for validating sensor calibration, where the sensor is allowed to deviate from its nominal value within a certain percentage. The detectability ($D_{EULM}$) for a sensor is expressed by the following formula:

(16) $$D_{EULM}=\;\frac{U}{\left(1-S_{A}\right)\times span}$$

where U is the expanded uncertainty of the model, $S_{A}$ is the sensor sensitivity value, and *span* is the sensor operational measurement range.

The advantage of the detectability metric is as follows. While the accuracy and sensitivity metrics allow for an overall assessment of model performance, they do not provide a specific indication of the smallest fault value that can be detected through the monitoring of prediction residual uncertainty [85].

## 6. Results and Discussion

This section outlines the parameters of the empirical models, estimated to represent the axial deformation dynamics in state-space and to implement the Kalman filter. The results of the performance evaluation of the predictive models, conducted during tests in the presence of outliers, measurement noise, and changes in the operating mode of the HU, are presented.

### 6.1. Predictive Models

Predictive models S1–S8 are the result of the modeling, verification, and optimization stages described in Section 2. They were determined using data on head pressure and axial deformation of the bolts, corresponding to datasets D1 and D2. Following the identification of the empirical models *Gi*(*s*), the verification stage revealed the presence of a slight negative bias in the predictions (see Table 3), with the greatest discrepancies observed during the system’s transient operation.

When applying the Kalman filter, the diagonal elements of the covariance matrix *Q* were set according to the relationship described in Section 3.3, using the maximum estimated uncertainty *U_MC_* for the models *Gi*(*s*), which corresponds to model S2 (see Table 3). The element of the covariance matrix *R* was set to the variance in sensor 7, as it represents the maximum axial deformation measurement variance among the eight sensors in dataset D2. The corresponding values are: *Q* = 0.008 and *R* = 0.061 (both in μm^2^).

Table 3 presents the parameters of the empirical models used for the state-space representation and the subsequent application of the Kalman filter to obtain the predictive models. The table also shows the accuracy metrics before and after applying the KF and the uncertainty associated with each model.

**Figure 5:**
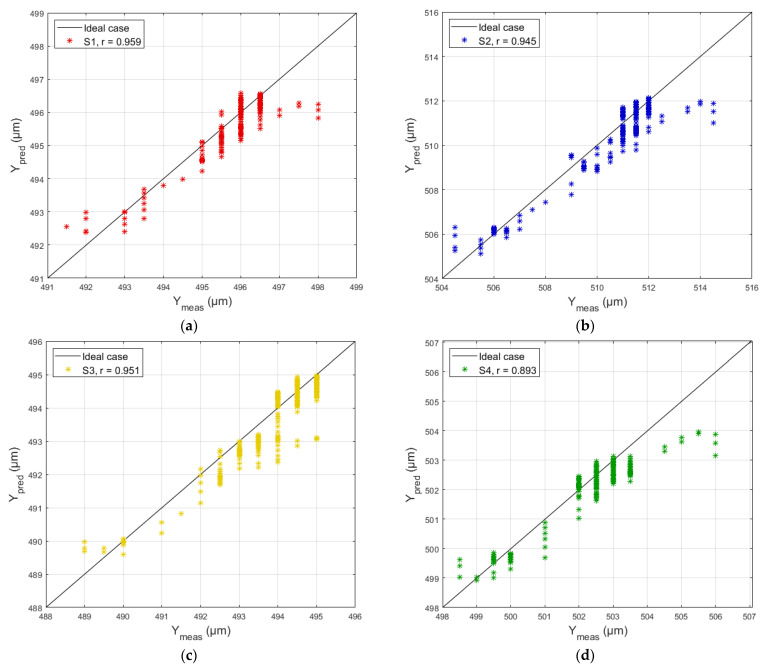

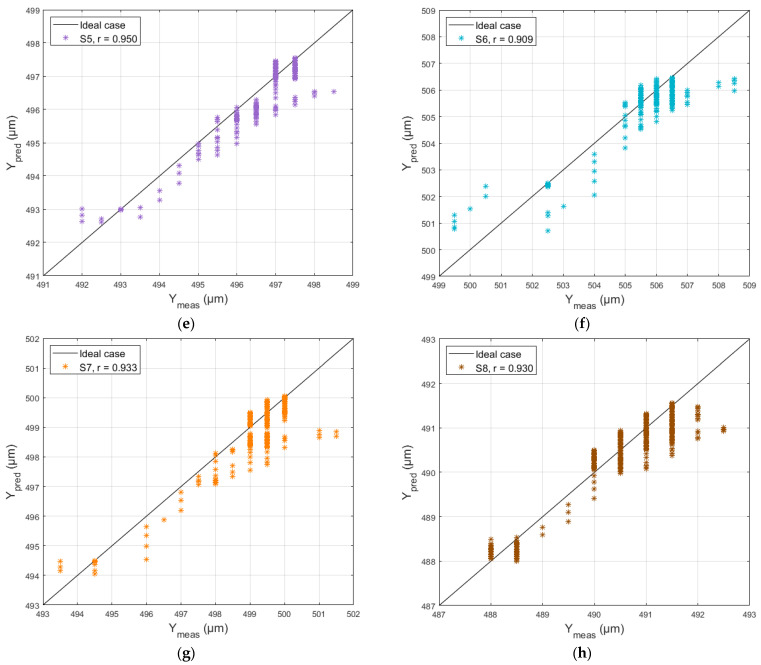
Correlation between the measured and predicted axial deformation values of the bolts. (**a**–**h**) show the correlation for each model S1–S8. The X-axis represents the measured values, and the Y-axis represents the predicted values (compiled by the authors).

From Table 3, it can be seen that the accuracy metrics (*Acc*) for predicting the axial deformation of the bolts, calculated before applying the Kalman filter (BFK), do not exceed a value of 0.672 µm. This value represents approximately 7% of the measurement system’s absolute error (see Table 1). After applying the filter (AFK), the maximum value of the accuracy metric decreases to 0.146 µm, which is equivalent to only 1.5% of the system’s absolute error. The uncertainties, estimated by the analytical method (*U_AM_*) and the Monte Carlo method (*U_MC_*), correspond to a 95% confidence interval (k = 2). The uncertainty *U_MC_* was estimated with *N* = 1000 simulations. Figure 5 shows the correlation between the measured and predicted values.

As can be seen in Figure 5, the Pearson correlation coefficient ‘*r*’ is close to 1, indicating a strong positive linear correlation. This confirms that the predicted values correspond to the measured ones.

### 6.2. Performance in the Presence of Outliers and Measurement Noise

As noted in Section 4.1, factors such as outliers and measurement noise can affect the predictive capability of the models. This subsection presents the test results for evaluating accuracy, sensitivity, and detectability under the influence of these factors. For calculating the sensitivity metric, in accordance with its defining Formula (15), both data containing deviations (outliers or noise) and data without deviations were used.

#### 6.2.1. Outlier Test

**Figure 6:**
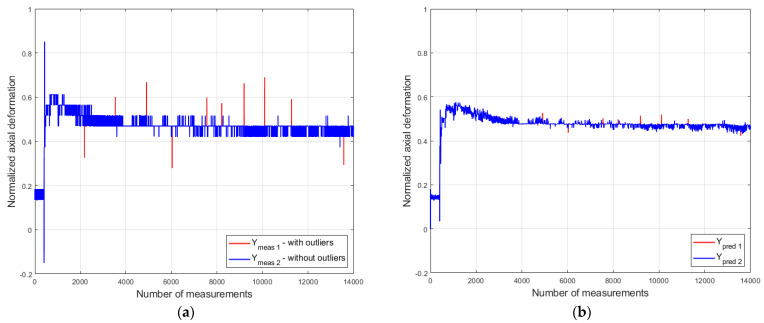
Model S2 test in the presence of outliers: (**a**) axial deformation data with and without outliers ($Y_{meas}$); (**b**) predicted axial deformation values ($Y_{pred}$) (compiled by the authors).

To evaluate the performance of the models in the presence of outliers, 10 outliers were randomly introduced into the axial deformation measurement data for each bolt from dataset D3. The simulated outlier values exceed the limit $x_{meas}+3\sigma$ specified in Section 4.1. The maximum σ value, calculated from the data of all eight sensors and amounting to 0.239 µm, corresponds to the measurements from sensor S7 in dataset D3. Figure 6 shows the test results in the presence of outliers, using model S2 as a representative example.

As can be seen from Figure 6b, the predictive model S2 mitigates the influence of outliers on the predicted axial deformation. Table 4 presents the performance metrics obtained in the presence of such outliers.

Despite the presence of outliers, the accuracy metric (*Acc*) reached a maximum value of 0.184 µm (model S8), which constitutes only 1.8% of the measurement system’s absolute error. In turn, the sensitivity metric ($S_{A}$) did not exceed the value of 0.0001 for any of the models. Regarding the fault detectability metric ($D_{EULM}$), calculated using the EULM method while accounting for prediction uncertainty, its value did not exceed 0.05% of the sensor’s operational measurement range. This means that the predictive models are capable of detecting fault values exceeding 0.25 µm by monitoring the uncertainty of the prediction residuals.

#### 6.2.2. Measurement Noise Test

**Figure 7:**
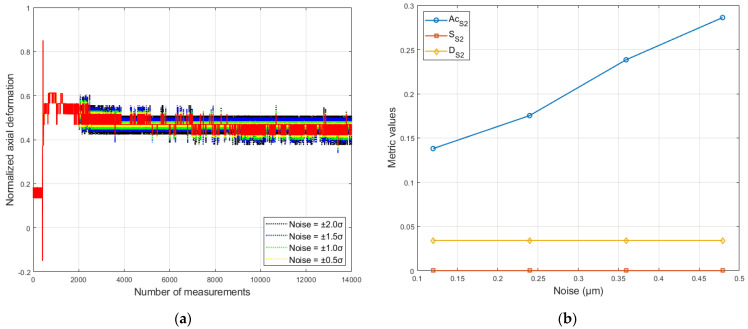
Model S2 test in the presence of measurement noise: (**a**) axial deformation data with different noise levels; (**b**) performance metrics under noise conditions (compiled by the authors).

Based on the definition of measurement noise provided in Section 4.1, the predictive capability of the models under its influence was evaluated. The simulated noise consisted of random values uniformly distributed within the ranges of ±0.5σ, ±1.0σ, ±1.5σ, and ±2.0σ. This allowed us to study its effect on the forecast by conducting four independent tests for each model. The simulated noise was added to the recorded deformation values of the bolts from dataset D3, starting from the 2000th measurement, as shown in Figure 7.

As can be seen from Figure 7b, the accuracy metric (*Acc*) exhibits an almost linear increase with the rise in simulated noise level. This pattern is observed for all analyzed models. On the other hand, the sensitivity ($S_{A}$) and detectability ($D_{EULM}$) metrics remain practically unchanged as noise increases. Table 5 provides a quantitative summary of these results, confirming the described patterns.

### 6.3. Performance Under a Change in the Operating Mode of the Hydroelectric Unit

**Figure 8:**
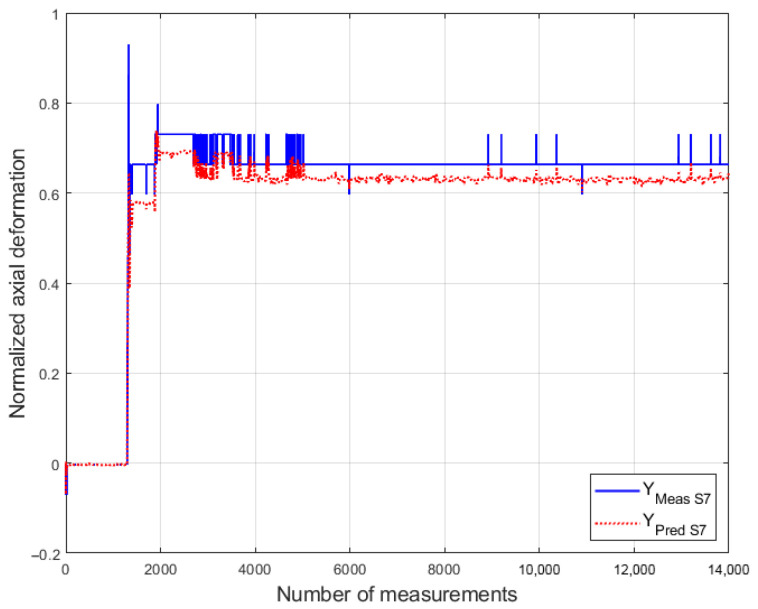
Measured and predicted axial bolt deformation values during a change in the operating mode of the HU for model S7 (compiled by the authors).

As explained in Section 4.1, it is necessary to verify the predictive capability of the models when the HU operates under a different mode (regime). This subsection evaluates the accuracy metric (*Acc*) of the predictive models under an operating regime at 70% of the nominal load, using data from the registration dataset D4. Figure 8 presents the measured bolt deformation values and the values predicted by model S7.

In turn, Table 6 presents the prediction accuracy metric (*Acc*) for each model, calculated using MAE, during a change in the HU operating mode.

### 6.4. Residual Behavior in the Presence of Faults

To study the models’ response to various types of faults, simulated anomalous values representing specific faults were artificially introduced into the axial bolt deformation data of dataset D3, starting from the 4000th measurement. For clear visualization, these anomalies are plotted alongside the measured values, which serve as a reference (baseline).

**Figure 9:**
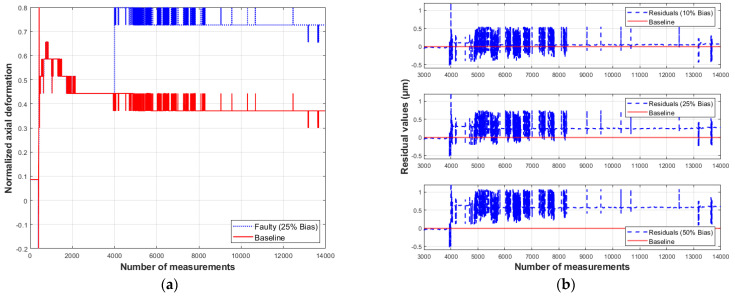
Response of model S1 to a bias-type fault: (**a**) measured and simulated values; (**b**) residual behavior (compiled by the authors).

**Figure 10:**
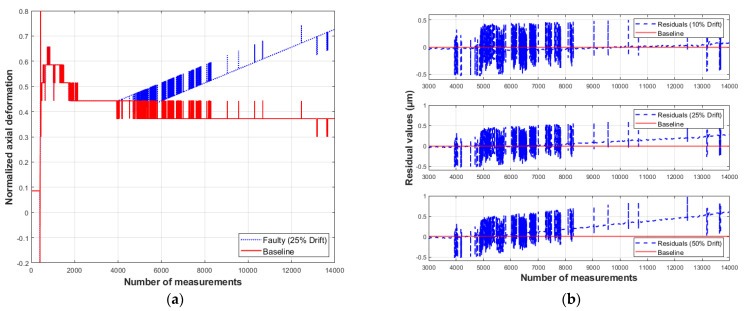
Response of model S1 to a drift-type fault: (**a**) measured and simulated values; (**b**) residual behavior (compiled by the authors).

**Figure 11:**
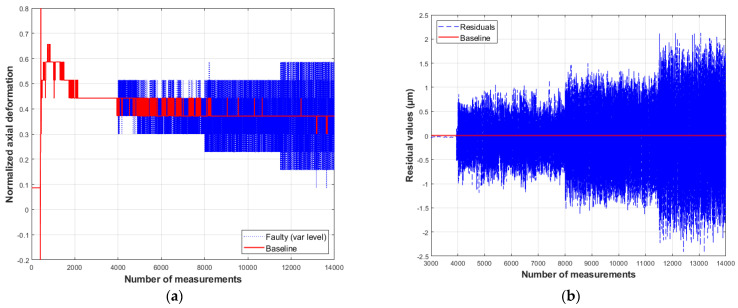
Response of model S1 to a precision degradation-type fault: (**a**) measured and simulated values; (**b**) residual behavior (compiled by the authors).

**Figure 12:**
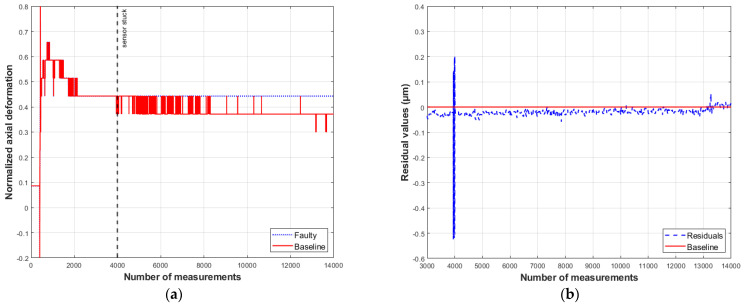
Response of model S1 to a stuck-at type fault: (**a**) measured and simulated values; (**b**) residual behavior (compiled by the authors).

On the residual plots, the baseline indicates no difference between the measured and predicted values. The residual vectors presented below illustrate the model’s response to the introduced anomalies (Figure 9, Figure 10, Figure 11 and Figure 12).

It is observed that a bias-type fault produces a peak at the moment of its occurrence. For a bias fault magnitude equal to 10% of the measurement system’s absolute error (equivalent to 1 µm), the residuals show virtually no deviation from the baseline. As the fault magnitude increases to 25% and 50%, the residual values deviate noticeably from the baseline. For a 50% fault, the change in the residual value is approximately 0.6 µm.

The results for the drift-type fault indicate that with a drift equivalent to 10% of the measurement system’s absolute error, the residuals remain close to the baseline without noticeable deviation. For a 25% drift (2.5 µm), beginning at the 4000th measurement, the residuals gradually deviate starting from the 8000th measurement, reaching ~0.3 µm. For a 50% drift, the deviation of the residuals begins at the 6000th measurement and reaches a maximum value of 0.6 µm.

The residual behavior during the simulation of a precision degradation in sensor 1 (Figure 10b) shows that the deviation of the residual values from the baseline increases proportionally to the fault severity. Specifically, when simulating an increase in measurement variance by 0.5, 1.0, and 1.5 µm, a corresponding rise in the residual value is observed, which is most significant at the maximum degradation level.

As seen in Figure 12a, the signal values of sensor S1 remain constant after the fault occurs. On the other hand, Figure 12b shows that residual behavior oscillates around the baseline after a transient period, without converging to zero. A peak is observed, caused by the change in axial deformation measurements at the moment of fault inception. Although the residual values are close to zero, they fluctuate randomly.

### 6.5. Discussion

According to the results obtained, the maximum value of the accuracy metric (*Acc*) achieved for the predictive models is 0.146 µm. This value corresponds to 1.5% of the measurement system’s absolute error, confirming the reliability of the predictions. Thus, the predictive models adequately describe the deformation dynamics of the turbine head-cover bolts under normal sensor operation during the HU power grid connection stage.

Tests conducted in the presence of outliers and measurement noise confirm the robustness of the models, as evidenced by sensitivity metric ($S_{A}$) values below 0.001. Detectability, assessed via the ($D_{EULM}$) metric, reaches a maximum value of approximately 0.01%. This result indicates that by monitoring residual uncertainty, it is possible to detect faults exceeding 0.05 µm.

Test conducted under changing operating mode of the HU showed an increase in prediction error, characterized by the accuracy metric (*Acc*). Although such an increase was expected, it is necessary to evaluate the predictive models under other operating modes. This would not only help determine the maximum prediction errors but also analyze their impact on the prediction uncertainty estimated for each model. Furthermore, these tests would allow for characterizing the maximum or variable prediction error depending on the operating mode, taking into account the uncertainty introduced by the dynamic operating conditions of the HU.

Based on the analysis of residual behavior under bias-type faults, it is evident that the moments of fault occurrence are evident; however, their magnitudes do not match the residual values. For example, according to Figure 9b, a fault equivalent to 50% of the measurement system’s absolute error (5 µm) results in a residual of about 0.6 µm. Regarding drift-type faults, the residual values not only fail to reflect the magnitudes of the introduced faults but also become distinguishable only after several measurements taken after their occurrence, as shown in Figure 10b. These results reveal a limitation of the proposed method, as for faults smaller than 50% of the system’s absolute error, the residuals are not easily distinguishable.

As previously indicated, the magnitudes of the simulated faults are not fully reflected in the residuals due to the partial compensation of the fault effect by the predictive models. More specifically, during the correction stage of the axial deformation estimation, the Kalman gain coefficient ($K_{k}$) attenuates the fault values based on the ratio of the covariance matrices Q (confidence in the dynamic model) and R (confidence in the actual observations). Therefore, the balance between both matrices could be optimized to improve the method’s sensitivity to lower-magnitude faults.

In contrast to the results for bias and drift faults, the residual signal for a fault caused by precision degradation exhibits distinct values corresponding to the simulated fault magnitude, which increase with the degradation level. Conversely, the residual values for a stuck-at fault do not converge to a constant value, as occurs with the sensor measurements. Instead, the residuals values fluctuate randomly with a small amplitude.

Although the predictive models S1–S8 partially compensate for the magnitude of faults when estimating bolt deformation, they generate characteristic residual patterns that allow for the identification of the fault type. These patterns can be integrated into fault detection and isolation (FDI) algorithms, enabling continuous and reliable monitoring of the sensors’ operational health. However, the fault detection time will be determined by the FDI algorithm, since each fault must be analyzed differently. We consider that, to ensure the reliability of axial deformation measurement results, this algorithm should include, among others, the following aspects:

- The various operating stages of the hydraulic unit and the changes in its operational loading modes;
- The definition of the maximum allowable deformation value for the fastening bolts;
- The permissible fault thresholds for the sensors before a failure state is considered;
- The analysis and estimation of measurement uncertainty sources, which will determine whether the sensors are within permissible thresholds.

## 7. Conclusions

This study proposes a method for ensuring the reliability of sensors’ measurement results in the system monitoring the axial deformation of hydraulic turbine head-cover fastening bolts. The method is based on analytical redundancy using predictive models. The main conclusions are as follows:

1. The error of the predictive models, evaluated by the accuracy metric, is approximately 2% of the measurement system’s absolute error. Therefore, the developed models adequately reflect the deformation dynamics of the fastening bolts, providing a foundation for their application in FDI algorithms.
2. The robustness of the predictive models was confirmed through testing under conditions involving outliers, measurement noise, and changes in the HU operating mode, as demonstrated by a sensitivity metric values below 0.001. Furthermore, the models are capable of detecting faults exceeding 0.05 µm by monitoring the uncertainty of the residuals.
3. The analysis of residual behavior demonstrated that the proposed analytical redundancy approach, based on the combination of empirical models and a Kalman filter, provides useful information for FDI. This enables not only the detection but also the identification of the sensor fault type, forming the basis for ensuring measurements results reliability.
4. Although the predictive models demonstrate accuracy and robustness, their validity has been confirmed only for the power grid connection stage when the HU operates under full and partial load (70%). Likewise, the residuals associated with bias-type and drift-type faults are difficult to discriminate when their magnitude is below 5 µm. Consequently, the application of this approach must be extended to the other operational stages of the HU. In addition, it is crucial to investigate which sensor fault types are most critical to monitor and to establish their corresponding threshold values.

## Figures and Tables

**Table 1:** Metrological characteristics of the axial deformation measurement system (compiled by the authors based on [54]).

Number of Measuring Channels	Displacement Measuring Range, µm	Absolute Error Limits, µm	Measuring Force, N	Scale Division, µm
8	from 0 to 10,000 *	±10	≤1.5	0.5

* The operational measurement range is from 250 to 760 µm.

**Table 2:** Sensor fault types (compiled by the authors).

Type of Fault	Definition	Causes	Ref.
Bias (Offset) fault	Constant deviation of the sensor-measured value from the true value across the entire or partial measurement range. The sensor’s output signal is offset by a fixed value.	Persistent calibration shift due to aging components, thermal stress, mechanical shock, or degradation of components within the signal processing circuitry.	[77,78,79]
Drift fault	Slow, progressive change in the characteristics of the sensor’s output signal over time, even when the value of the measured physical quantity remains constant.	Aging of electronic components, chemical degradation of the sensing element, contamination, etc.	[80,81]
Precision Degradation (Noise) Fault	Increase in the variance in the sensor output signal, leading to a loss of resolution and precision, even if the signal’s mean value remains correct.	Degradation of electrical contacts, electromagnetic interference, component overheating, poor-quality power supply caused by increased voltage variance.	[79,82,83]
**Stuck-at fault**	The sensor output signal becomes fixed at a constant value, and the sensor ceases to respond to input stimuli.	Sensor wire break, short circuit to the supply line, failure of the sensing element, malfunction of the analog-to-digital converter, inadmissible increase in sensor friction.	[79,84]

**Table 3:** Parameters of the empirical models, their uncertainties, and accuracy metrics (compiled by the authors).

Parameter	Model S1	Model S2	Model S3	Model S4	Model S5	Model S6	Model S7	Model S8
*Kp*	2.240	2.549	3.571	1.805	3.134	2.163	3.207	2.462
*Tz*	−2.520	−3.806	−0.133	−5.106	−1.358	−3.029	−0.498	−0.857
*Tp1*	32.264	35.212	33.065	29.116	33.647	32.395	33.373	25.879
*Tp2*	0.946	0.966	0.853	0.952	0.946	0.913	0.921	0.948
**Uncertainty**								
*U_AM_*, μm	0.097	0.117	0.125	0.101	0.114	0.103	0.122	0.108
*U_MC_*, μm	0.128	0.174	0.168	0.145	0.170	0.144	0.172	0.143
**Bias and Accuracy**								
*Bias*, μm	−0.456	−0.623	−0.463	−0.164	−0.230	−0.559	−0.426	−0.421
*Acc* BFK, μm	0.457	0.672	0.479	0.491	0.489	0.561	0.582	0.550
*Acc* AFK, μm	0.036	0.134	0.103	0.146	0.103	0.090	0.132	0.107

**Table 4:** Performance metrics of the models in the presence of outliers (compiled by the authors).

Metric	Model S1	Model S2	Model S3	Model S4	Model S5	Model S6	Model S7	Model S8
*Acc*, μm	0.112	0.151	0.108	0.155	0.069	0.140	0.135	0.184
*S_A_*	1.00 × 10^−4^	1.00 × 10^−4^	1.00 × 10^−4^	1.00 × 10^−4^	1.00 × 10^−4^	1.00 × 10^−4^	1.00 × 10^−4^	1.00 × 10^−4^
*D_EULM_*, %	0.025	0.034	0.033	0.028	0.033	0.028	0.034	0.028

**Table 5:** Performance metric results of the models in the presence of measurement noise (compiled by the authors).

Metric	Noise Level	Model S1	Model S2	Model S3	Model S4	Model S5	Model S6	Model S7	Model S8
*Acc*, μm	0.5σ	0.114	0.139	0.106	0.096	0.096	0.139	0.134	0.181
*Acc*, μm	1.0σ	0.154	0.176	0.149	0.144	0.144	0.173	0.172	0.204
*Acc*, μm	1.5σ	0.221	0.239	0.218	0.217	0.217	0.236	0.234	0.255
*Acc*, μm	2.0σ	0.271	0.284	0.267	0.266	0.264	0.281	0.280	0.297
* *S_A_* mean	-	2.57 × 10^−5^	2.57 × 10^−5^	2.42 × 10^−5^	2.56 × 10^−5^	2.56 × 10^−5^	2.37 × 10^−5^	2.52 × 10^−5^	2.49 × 10^−5^
* *D_EULM_* mean, %	-	0.025	0.034	0.033	0.028	0.033	0.028	0.034	0.028

* Shows the mean values of sensitivity and detectability for the predictive models.

**Table 6:** Accuracy metric results of the models during operation at 70% of nominal load (compiled by the authors).

Metric	Model S1	Model S2	Model S3	Model S4	Model S5	Model S6	Model S7	Model S8
*Acc*, μm	0.213	0.333	0.312	0.251	0.243	0.405	0.266	0.236

## Data Availability

The normalized datasets necessary to reproduce the key results of this study are available from the corresponding authors upon reasonable request. Further raw data and supporting datasets cannot be made publicly available due to legally binding commercial confidentiality agreements with the industrial partner that owns and operates the turbine. Any shared data will be limited to non-commercial academic research purposes and require a formal data sharing agreement.

## Abbreviations

SC	Self-Calibration
OLM	On-Line Monitoring
MSC	Metrological Self-Control
SEVA	Self-Validating Sensor Technology
KF	Kalman Filter
MSE	Mean Squared Error
EUML	Error Uncertainty Limit Monitoring
GUM	Guide to the Expression of Uncertainty in Measurement
FDI	Fault Detection and Identification
